# Needs and Barriers of Mental Health Professionals in Promoting Smoking Cessation

**DOI:** 10.1192/j.eurpsy.2025.980

**Published:** 2025-08-26

**Authors:** L. Pérez De Lucia Bové, S. Ravelo, C. Bountzas, Y. Khazaal

**Affiliations:** 1 CHUV, Lausanne; 2 CNP, Neuchâtel, Switzerland

## Abstract

**Introduction:**

Various studies have shown that individuals with mental health disorders are significantly more likely to smoke compared to the general population. Additionally, they tend to exhibit more severe tobacco dependence. For these populations, smoking is associated with increased morbidity, mortality, and healthcare costs. Despite the significance of this issue and its impact on health, quality of life, and financial well- being, smoking cessation interventions are rarely implemented in mental health services.

**Objectives:**

The STEN project (alleviate Stigma, Train, Enhance smoking cessation interventions, rely on a specialized Network), funded by the Tobacco Prevention Fund in Switzerland, aims to promote smoking cessation among individuals with mental health disorders. The project seeks to strengthen the competencies of mental health professionals, encourage the utilization of available resources, and shift healthcare professionals’ beliefs about smoking in individuals with mental disorders.

**Methods:**

The first phase of the STEN project focuses on identifying the needs and perceptions of mental health professionals regarding smoking cessation. This phase involves a qualitative opinion survey exploring the perceived barriers and needs related to treating tobacco dependence in people with mental health disorders. The survey was conducted through focus groups held between December 2023 and April 2024, involving about 80 professionals from various linguistic regions of Switzerland, representing different professional bodies and both private and public addiction and psychiatry services. Data collection was facilitated and standardized using a structured response document.

**Results:**

The analysis of the focus group discussions revealed:

Barriers: The main barriers to implementing tobacco dependence treatment among mental health professionals included a lack of knowledge, the misconception that addressing tobacco use is not part of their mission, and false beliefs about patients’ willingness to quit.

Needs: There is a strong interest among mental health professionals in digital training, accompanied by opportunities for practice-sharing.

**Conclusions:**

This phase will culminate in the development of a training and digital support concept tailored to the needs and perceptions of mental health professionals in Switzerland.

**Disclosure of Interest:**

None Declared

